# A Metabolomic Signature of Ischemic Stroke Showing Acute Oxidative and Energetic Stress

**DOI:** 10.3390/antiox13010060

**Published:** 2023-12-29

**Authors:** Moustapha Djite, Juan Manuel Chao de la Barca, Cinzia Bocca, Ndiaga Matar Gaye, Néné Oumou Kesso Barry, Mame Ndoumbé Mbacke, Ousmane Cissé, Pape Matar Kandji, Ndèye Marème Thioune, Najah Fatou Coly-Gueye, El Hadji Malick Ndour, Fatou Gueye-Tall, Amadou Gallo Diop, Gilles Simard, Delphine Mirebeau-Prunier, Papa Madieye Gueye, Pascal Reynier

**Affiliations:** 1 Laboratoire de Biochimie Pharmaceutique, Faculté de Médecine, Pharmacie, Université Cheikh Anta Diop, Dakar 2238, Senegal; oumou.barry22@yahoo.com (N.O.K.B.); elhadjimalickndour@yahoo.fr (E.H.M.N.); fatougueye82@yahoo.fr (F.G.-T.); papamadieye.gueye@ucad.edu.sn (P.M.G.); 2 Laboratoire de Biochimie, Centre Hospitalier National Universitaire (CHNU) de FANN, Dakar 45701, Senegal; farima9036@gmail.com (M.N.M.); kandjipapematar@gmail.com (P.M.K.); thiounemaria09@gmail.com (N.M.T.); 3 Département de Biochimie et Biologie Moléculaire, Centre Hospitalier Universitaire (CHU), 49933 Angers, France; jmchaodelabarca@chu-angers.fr (J.M.C.d.l.B.); cinzia.bocca@univ-angers.fr (C.B.); gisimard@chu-angers.fr (G.S.); deprunier@chu-angers.fr (D.M.-P.); pareynier@chu-angers.fr (P.R.); 4 Unité Mixte de Recherche (UMR) MITOVASC, Institut National de la Santé et de la Recherche Médicale (INSERM U-1083), Centre National de la Recherche Scientifique (CNRS 6015), Université d’Angers, 49933 Angers, France; 5 Clinique Neurologique, Centre Hospitalier National Universitaire (CHNU) de FANN, Dakar 45701, Senegal; ndiagamatar@gmail.com (N.M.G.); ouscis01@hotmail.fr (O.C.); agallodiop@gmail.com (A.G.D.); 6 Hôpital Pour Enfants de Diamniadio, Dakar 10700, Senegal; najahfatoucoly@yahoo.fr

**Keywords:** cryptogenic ischemic stroke, energetic metabolism, metabolomics, lipidomics, mitochondria, oxidative stress, stroke

## Abstract

Metabolomics is a powerful data-driven tool for in-depth biological phenotyping that could help identify the specific metabolic profile of cryptogenic strokes, for which no precise cause has been identified. We performed a targeted quantitative metabolomics study in West African patients who had recently suffered an ischemic stroke, which was either cryptogenic (*n* = 40) or had a clearly identified cause (*n* = 39), compared to a healthy control group (*n* = 40). Four hundred fifty-six metabolites were accurately measured. Multivariate analyses failed to reveal any metabolic profile discriminating between cryptogenic ischemic strokes and those with an identified cause but did show superimposable metabolic profiles in both groups, which were clearly distinct from those of healthy controls. The blood concentrations of 234 metabolites were significantly affected in stroke patients compared to controls after the Benjamini–Hochberg correction. Increased methionine sulfoxide and homocysteine concentrations, as well as an overall increase in saturation of fatty acids, were indicative of acute oxidative stress. This signature also showed alterations in energetic metabolism, cell membrane integrity, monocarbon metabolism, and neurotransmission, with reduced concentrations of several metabolites known to be neuroprotective. Overall, our results show that cryptogenic strokes are not pathophysiologically distinct from ischemic strokes of established origin, and that stroke leads to intense metabolic remodeling with marked oxidative and energetic stresses.

## 1. Introduction

Stroke is a leading cause of death and disability worldwide. In Africa, stroke incidence and prevalence rates are estimated to be up to 2 to 3 times higher than in Western Europe and the United States. An annual incidence rate of up to 316 per 100,000 individuals, a prevalence of up to 1460 per 100,000, and a 3-year case fatality rate of more than 80% have been estimated [1]. Strokes in African populations tend to be more severe with a higher mortality rate than in other populations [2]. Stroke survivors in African populations tend to have a higher disability burden, with higher rates of post-stroke depression, cognitive impairment, and physical disability [3]. Stroke tends to occur at a younger age in African populations, with a peak incidence in the 45- to 54-year age range [3]. Hypertension, which constitutes a major risk factor for stroke, is also more common in Africans than in other populations [4]. In Senegal, stroke is the leading neurological disease and accounts for 1/3 of hospitalizations and 2/3 of deaths in the Neurology Department of the FANN University Hospital of Dakar [5].

Cryptogenic stroke is defined as “a stroke that is not caused by cardiac or arterial embolic sources, small vessel occlusion, or other identifiable cause despite a standard diagnostic evaluation” [6]. Cryptogenic stroke is thus diagnosed when all other potential causes of a stroke have been ruled out through medical imaging tests, such as CT (computed tomography) or MRI (magnetic resonance imaging) scans, blood tests, and heart tests. Cryptogenic stroke may account for approximately 30–40% of all ischemic strokes [7]. Although the exact causes of cryptogenic stroke are unknown, it is thought that they may be linked to underlying conditions, such as undiagnosed brain, heart, or clotting damage.

The development of clinical metabolomics, particularly the more standardized quantitative targeted metabolomics, provides an opportunity to explore the deep biological phenotype of diseases. The mechanisms and risk factors leading to ischemic stroke can be multiple, with heterogeneous clinical consequences depending on the affected regions of the brain and the extent of these. Metabolomics offers the possibility to stratify these different causes and consequences of stroke. Metabolomic stratification could facilitate greater personalization of the diagnostic classification, management, and treatment of patients. Many metabolomic studies have investigated metabolic remodeling in the blood of stroke patients. They consistently showed metabolic disturbances, such as alteration of one-carbon and energetic metabolism, oxidative stress, glutamate excitotoxicity, neuroinflammation, and lipid remodeling [8,9,10]. Overall, these findings show that stroke is a complex disease involving widespread metabolic disruptions.

No previous metabolomic studies related to stroke have been reported in African patients to our knowledge, and it is difficult to identify studies that have looked for specific altered metabolomic profiles in cryptogenic ischemic stroke. We present here a targeted quantitative metabolomics and lipidomics study performed in patients from the National University Hospital of FANN in Dakar. The primary objective of this study was to delineate the metabolomic profile specific to cryptogenic strokes compared to strokes with a known etiology. The secondary objective was to characterize the whole metabolomic signature of the two forms of stroke compared with the healthy control group.

## 2. Materials and Methods

### 2.1. Ethic Statements

This study was approved by the Research Ethics Committee (REC) of Cheikh Anta Diop University (UCAD), in accordance with the rules set forth by the National Health Research Ethics Committee (CNERS) of Senegal under the number 0412/2019/CER/UCAD.

### 2.2. Study Participants

This was a prospective case–control study of subjects who had suffered a cryptogenic stroke compared with two groups of controls: one group of patients who had suffered a recent stroke whose etiology was well identified and one group of healthy controls. Participants were consecutively recruited from the Neurology Department of the FANN National University Hospital in Dakar between 1 June 2019 and 30 April 2021, with a break during the COVID-19 pandemic. Biochemical parameters were measured at the Biochemistry Laboratory of the FANN National University Hospital in Dakar. The metabolomic study was retrospectively performed on the collected blood samples at the Department of Biochemistry and Molecular Biology of the University Hospital of Angers, France.

**Figure 1:**
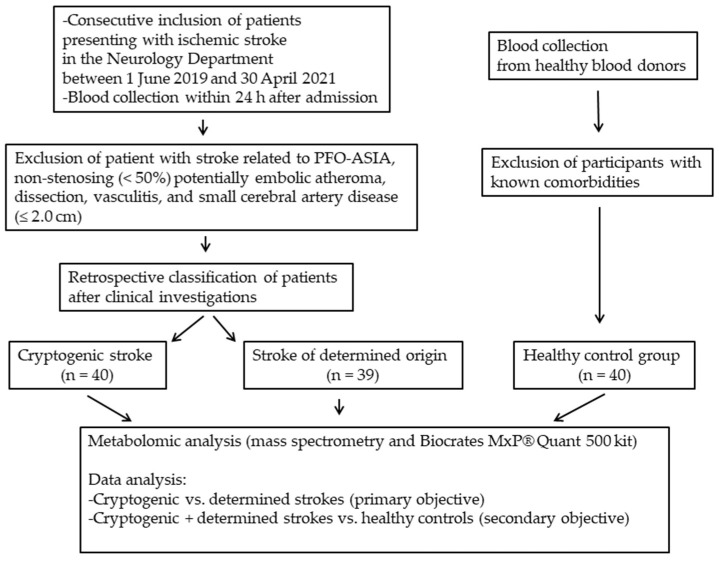
Study flowchart.

Forty subjects who had suffered a recent cryptogenic stroke were included according to the TOAST (Trial of Org 10172 in Acute Stroke Treatment) classification [11] (Figure 1). These cryptogenic strokes were diagnosed on clinical grounds and confirmed by the absence of abnormalities in the cerebral CT scan, the absence of high-risk emboligenic heart disease, and extra- and intra-cranial stenosing atheroma (>50%). Subjects who had suffered a stroke related to PFO-ASIA (Permeable Foramen Ovale–Aneurysm of the Inter-Arial Septum), with non-stenosing (<50%) potentially embolic atheroma, dissection, vasculitis, or small cerebral artery disease (≤2.0 cm), were excluded from the study.

Two groups of control subjects were recruited. Blood samples were taken under the same pre-analytical conditions as for the subjects who had suffered a cryptogenic stroke. The first group of control subjects consisted of 39 subjects with ischemic stroke of determined cause, who were monitored in the same Neurology Department. The second group of control subjects consisted of 40 healthy subjects recruited from among blood donors. All participants with clearly identified comorbidities, such as diabetes, dyslipidemia, hypertension, cardiovascular and metabolic diseases, and cancer, were excluded from this healthy control group.

### 2.3. Studied Parameters

Epidemiological and clinical parameters were recorded for all participants, including diabetes, which was determined according to the 2006 WHO criteria (https://www.who.int/news-room/fact-sheets/detail/diabetes (accessed on 15 January 2023)), as well as physical inactivity (https://www.who.int/news-room/fact-sheets/detail/physical-activity (accessed on 15 January 2023)). Smoking and alcohol consumption were determined based on a questionnaire and according to the answers given by the patients. Chronic alcoholism was considered in men who consumed five glasses per day, i.e., 50 g of ethanol per day, and in women who consumed three glasses per day, i.e., 30 g of ethanol per day. For smoking, a threshold of five cigarettes per day was defined, i.e., 50 ug of nicotine absorbed per day. The routine biochemical parameters were determined using an Architect ci4100 (Abbott, Abbott Park, IL, USA) using enzymatic techniques in accordance with the manufacturer’s recommendations.

### 2.4. Blood Sample Collection

Blood samples were taken within 24 h of admission to the Neurology Department in the morning after 12 h of fasting. Blood was collected in heparin tubes that were immediately transported on ice and centrifuged for 5 min at 3000× *g* at +4 °C. Aliquots of 50 µL of the supernatant (heparinized plasma) were prepared in 5 cryotubes and stored at −80 °C. They were then transported in dry ice to the Biochemistry Laboratory of the University Hospital of Angers, France, where metabolomic analysis was performed.

### 2.5. Metabolomic Analysis

A targeted quantitative metabolomics analysis was performed using the MxP^®^ Quant 500 kit (Biocrates Life Sciences AG, Innsbruck, Austria). When used with mass spectrometry (QTRAP 5500; SCIEX, Villebon-sur-Yvette, France), this kit allows the quantification of up to 630 endogenous metabolites from 26 biochemical classes, including alkaloids (*n* = 1), amines oxide (*n* = 1), amino acids (*n* = 20), amino acids related (*n* = 30), bile acids (*n* = 14), biogenic amines (*n* = 9), carbohydrates comprising the sum of hexoses (*n* = 1), carboxylic acids (*n* = 7), cresols (*n* = 1), fatty acids (*n* = 12), hormones and related (*n* = 4), indoles and derivatives (*n* = 4), nucleobases and related (*n* = 2), vitamins and cofactors (*n* = 1), acylcarnitines (*n* = 40), lysophosphatidylcholines (*n* = 14), phosphatidylcholines (*n* = 76), sphingomyelins (*n* = 15), ceramides (*n* = 28), dihydroceramides (*n* = 8), hexosylceramides (*n* = 19), dihexosylceramides (*n* = 9), trihexosylceramides (*n* = 6), cholesteryl esters (*n* = 22), diglycerides (*n* = 44), and triglycerides (*n* = 242). Flow injection analysis coupled with high-resolution mass spectrometry (FIA-MS/MS) was used to analyze lipids and hexoses. Liquid chromatography (LC-MS/MS) was used to separate the other polar metabolites according to their retention time. Two MxP^®^ Quant 500 kits were used to quantify the sample metabolome, as one kit has room for up to 80 samples.

All reagents used in this analysis were of LC-MS quality and purchased from VWR (Fontenay-sous-Bois, France) and Merck (Molsheim, France). Sample preparation and analysis were performed according to the Biocrates kit user manual. Each plasma sample was carefully vortexed after thawing and centrifuged at +4 °C for 5 min at 5000× *g*. After this, 10 microliters of each sample were added to the upper well filter of the 96-well plate. The metabolites were extracted and derivatized for quantification of amino acids and biogenic amines. Finally, extracts were diluted with the MS operating solvent prior to analysis by FIA and LC-MS/MS. Three quality controls (QCs) consisting of human plasma samples at three concentration levels—low (QC1), medium (QC2), and high (QC3)—were used to evaluate the performance of the analytical assay. A seven-point serial dilution of the calibrators was added to the 96-well kit plate to generate calibration curves for the quantification of amino acids and biogenic amines. As an additional quality control, a pool consisting of a mixture of several samples from the three compared groups was run 3 times on each kit.

### 2.6. Data Analyses

After validation of the quality controls and exclusion of metabolites that were not correctly assayed, the kit (i.e., batch) effect was removed using the R software limma package (version 16.0) [12]. Multivariate analyses were performed using SIMCA^®^ 17.0 software (Umetrics, Umea, Sweden). Principal component analysis (PCA) was performed on each group separately to study the overall structure of the data. Under the hypothesis of multivariate segregation between control and stroke plasma samples, it is not appropriate to mix both populations to eliminate potential outliers using a single PCA. In the latter case, it is better to carry out a PCA on controls and then possibly eliminate samples that behave differently (i.e., outliers) compared to other samples in this group. The process was then carried out separately for the stroke samples. Once potential outliers were eliminated, the remaining samples were used in the univariate and multivariate analyses and put together for a common PCA to detect groups of similar samples. It is worth noting that performing PCA on each group separately does not create between-group differences but was only performed to eliminate samples that did not fit in their own group. After this, a supervised OPLS-DA analysis (orthogonal partial least square discriminant analysis) was performed. OPLS-DA works similarly to PCA but aims at maximizing discrimination between compared groups. The parameter used to measure the predictive ability of the models is called Q^2^Y_cum_, which is the complement of the prediction error sum of squares relative to the total sum of squares. A threshold of 0.5 is set to decide whether the model found has a good predictive capability (i.e., Q^2^Y_cum_ ≥ 0.5, meaning the model better discriminates between compared groups than a random model). In addition to Q^2^Y_cum_, the *p*-value of the CV-ANOVA (cross-validated-analysis of variance) test (*p*-value_CV-ANOVA_) and the Q^2^Y_cum_ obtained after totally permuting the entries of the Y vector whilst keeping the rows and columns of the matrix of predictive variables (i.e., metabolites) unchanged or Q^2^Y_cum,perm_ are common parameters used to evaluate model overfitting. Models explaining data much better than chance have a *p*-value_CV-ANOVA_ ≤ 0.05 and a negative realization of Q^2^Y_cum,perm_. If a discriminating low-overfitted model is obtained, the weight of each metabolite in the model construction is measured by its loading (p) on the predictive latent variable (pLV). The projection (loading) on the pLV of each variable in the original data set (i.e., each metabolite) is measured as a correlation coefficient (p_corr_) between the metabolite and the pLV.

Student’s *t*-test was used to compare metabolite concentrations between each group. The Benjamini–Hochberg correction was applied to correct for type 1 error inflation and to keep the false-discovery rate below 5%. Metabolite selection was ultimately based on two pieces of information put together in a graph called a volcano plot: p_corr_ from multivariate analysis and *p*-values from univariate analysis. The statistical analysis of sums and ratios of metabolites was carried out using the non-parametric Mann–Whitney–Wilcoxon test.

## 3. Results

### 3.1. Clinical Features

The causes of stroke in the strokes of the definite origin control group were ischemic in 100% of cases, with a thromboembolic cause in 44% of patients, small-vessel involvement (lacunar ischemia) in 33%, and atherothrombotic etiology in 23%.

The mean age of the cryptogenic ischemic stroke group (47 years) was 10 years younger than that of the established-origin ischemic stroke group (57 years, *p* < 0.001) but similar to that of the healthy control group (47 years) (Table 1). The sex ratio (1.35) was similar between the three groups. According to the biological analyses, the cryptogenic stroke group had significantly lower blood levels of glucose, urea, C-reactive protein, AST, and alkaline phosphatase compared with the established-origin stroke control group. Lipids, creatinine, and other liver enzymes were comparable between the two groups. On a clinical level, the cryptogenic stroke group was significantly less affected by hypertension, diabetes, and history of dyslipidemia than the control group of strokes of determined origin. However, there was significantly greater smoking, alcohol, and drug use within the cryptogenic stroke group than in the established-origin stroke control group.

### 3.2. Metabolomic Analysis

**Figure 2:**
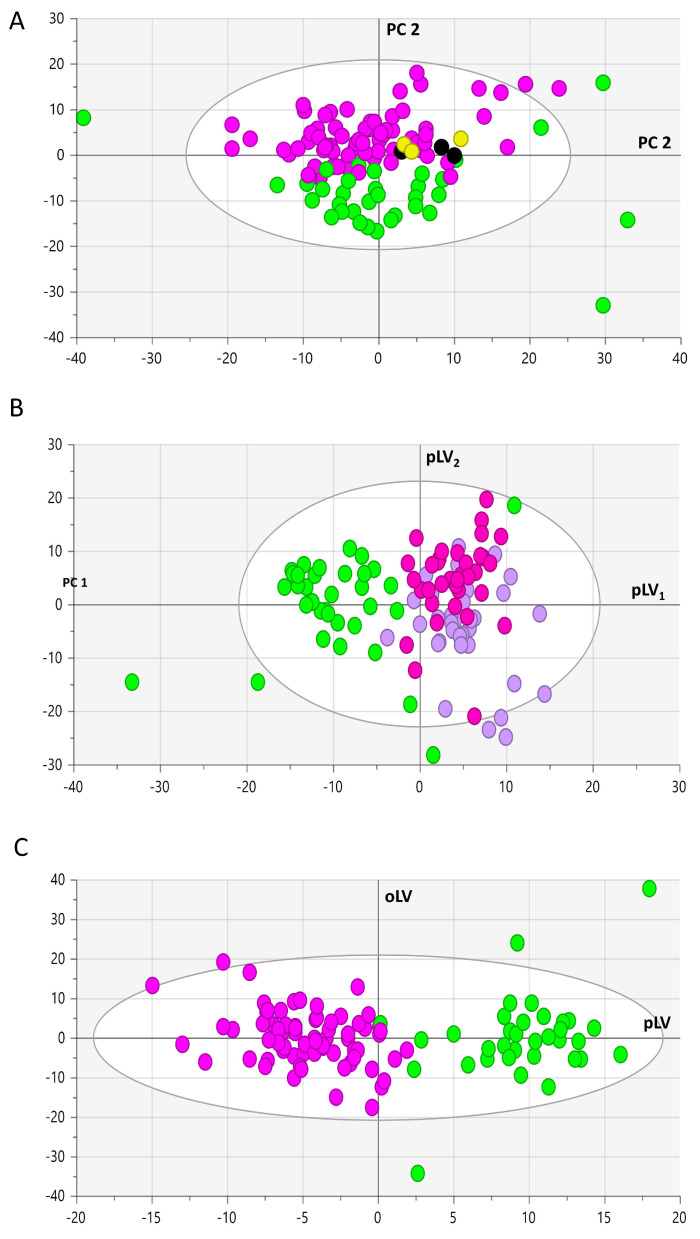
Principal component analysis (PCA) and orthogonal projection to latent structures discriminant analysis (OPLS-DA) scatter plot with samples represented as circles. (**A**) The first plan of the principal component analysis including samples from controls (green), stroke (violet), and pool samples. Pool samples analyzed in the first batch are colored in yellow and those analyzed in the second batch are colored in black. There is a neat distinction between samples from controls and stroke patients in the second principal component (PC2) whilst pool samples plot near the origin (0,0) with no clear batch effect. (**B**) OPLS-DA analysis of all samples aiming at discriminating between controls (green), cryptogenic strokes (light violet), and strokes of known origin (dark violet), which are considered as three different groups. This model had no good predictive capabilities mainly due to the lack of discrimination between cryptogenic strokes and strokes of a known etiology. (**C**) However, as expected from the PCA analysis, there is a clear distinction between control (green circles) and stroke (dark violet circles)samples taken as a single group. OPLS-DA scatter plot PCA and OPLS-DA axes are dimensionless. Legend: PC1, PC2: first and second principal components; pLV_1_, pLV_2_: first and second predictive latent variables; pLV: predictive latent variable; oLV: orthogonal latent variable.

Of the 630 metabolites analyzed, 456 were considered accurately measured. The raw data (56,986 plasma concentrations in μM of 456 metabolites from 119 participants and pools) are presented in Supplementary Table S1. The PCA showed a marked batch effect (Supplementary Figure S1A) that disappeared after batch correction (Supplementary Figure S1B). PCA identified 6, 4, and 5 outliers in the control group, stroke-of-known-origin group, and strokes of the cryptogenic-origin group, respectively. These samples were eliminated from further statistical analyses. PCA of all remaining samples and pool samples showed a marked grouping of stroke samples compared to control samples (Figure 2A). The OPLS-DA algorithm failed at separating strokes of unknown origin from those with a well-identified origin when all groups were put in the same model (Figure 2B). However, control samples were well separated from the group of stroke samples independently of their known or unknown origin (Figure 2C). This OPLS-DA model separating all the strokes from the controls showed good predictive capabilities and a low risk of overfitting (Q^2^Y_cum_ = 0.66; *p*-value_CV-ANOVA_ = 2.08096 × 10^−22^; Q^2^Y_cum-perm_ = −0.34).

Univariate analysis showed 234 metabolites with significantly different mean concentrations between stroke (unknown and known origin) and control samples with a false-discovery rate of 0.047.

**Figure 3:**
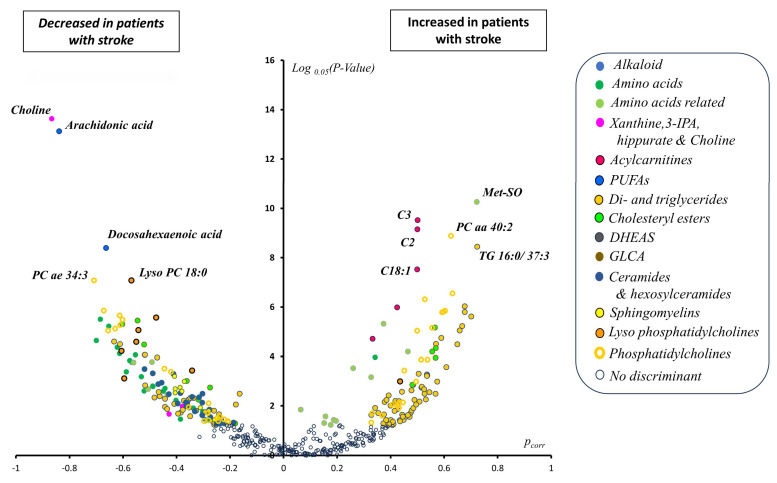
Volcano plot combining uni- and multivariate analyses. The *x*-axis represents loadings from the OPLS-DA analysis discriminating between control and stroke patients, with positive/negative values indicating relatively elevated/diminished concentration in the plasma of stroke patients compared to that of controls. Log-transformed *p*-values obtained from the comparison between both groups form the *y*-axis, using 0.05 as the base for logarithm calculation. Only metabolites that retained their significance after the Benjamini–Hochberg correction are displayed as colored circles. Some significant metabolites are also labeled due to very low (i.e., very significant) *p*-values and were positively correlated (methionine sulfoxide (Met-SO), acetyl (C2), propionyl (C3) and oleoyl (C18:1) carnitines, diacyl phosphatidylcholine PC aa 40:2, and triacylglyceride TG 16:0/37:3) or negatively correlated (choline, arachidonic acid, docosahexaenoic acid, lysophosphatidylcholine 18:0, and acyl-alkyl phosphatidylcholine PC ae 34:3) with the predictive latent variable. Legend: *P_corr_*: loading of each metabolite on the predictive latent variable represented as a correlation coefficient; 3-IPA: 3-indolepropionic acid; PUFAs: polyunsaturated fatty acids as acyl moieties in phosphatidylcholine species; DHEAS: dehydroepiandrosterone sulfate; GLCA: glycolithocholic acid.

The most important metabolites based on loadings (measured as correlation coefficients between metabolites and the predictive latent variable of the OPLS-DA model or pcorr) and corrected log-transformed *p*-values (using 0.05 as the base of the logarithm) are displayed in Figure 3. The corresponding *p*-values and loadings, as well as fold changes, are given in Supplementary Table S2.

The *p*-values and fold changes (FC, stroke/controls) of the most discriminant metabolites shown in Figure 3 were methionine sulfoxide (*p* = 4.54 × 10^−14^; FC = 2.7), C2 (*p* = 1.24 × 10^−12^; FC = 2.8), C3 (*p* = 4.12 × 10^−13^; FC = 2.0), C18:1 (*p* = 1.62 × 10^−10^; FC = 1.6), PC aa 40:2 (*p* = 2.68 × 10^−12^; FC = 2.1), and TG 16:0/37:3 (*p* = 1.038 × 10^−11^; FC = 2.5) for the increased metabolites and choline (*p* = 1.84 × 10^−18^; FC = 0.07), arachidonic acid (*p* = 8.36 × 10^−18^; FC = 0.36), docosahexaenoic acid (*p* = 1.22 × 10^−11^; FC = 0.34), LysoPC18:0 (*p* = 6.17 × 10^−10^; FC = 0.56), and PC ae 34:3 (*p* = 6.07 × 10^−10^; FC = 0.47) for the decreased metabolites. The *p*-value and FC of all other metabolites are available in Supplementary Table S2. According to these analyses, variations of top metabolites grouped into their biochemical family and taking the stroke group as a reference compared to controls were as follows:

(1) Diminished alkaloid trigonelline.
(2) Diminished non-proteinogenic (ornithine and citrulline) and proteinogenic amino acids (save cysteine with irrelevant variation).
(3) Amino-acid-related molecules: increased levels of methionine sulfoxide, homocysteine, dopamine, serotonin, GABA, α-aminobutyrate, α-aminoadipate, 1-methylhistidine, and polyamines (putrescine, spermidine, and spermine) and decreased levels of betaine, pro-betaine, tryptophane-betaine, sarcosine, and β-alanine.
(4) Other polar metabolites: decreased levels of choline, hippurate, 3-indolepropionate, and xanthine.
(5) Acylcarnitines: increased levels of short-chain acetyl (C2), propionyl (C3), and butyryl (C4) carnitines and long-chain oleoyl (C18:1) and linoleyl (C18:2) carnitines.
(6) Free or non-esterified fatty acids: diminished concentration of arachidonic (AA or FA 20:4 ω6) and docosahexaenoic (DHA or FA 22:6 ω3) acids.
(7) Acylglycerols: diminished concentration of 3 diacylglycerols and increased concentration of 1 diacylglycerol molecule and decreased concentration of 28 triacylglycerol (TAG) species and increased concentration of 57 TAG molecules. Diminished TAGs were mainly polyunsaturated whilst the opposite happened for the increased TAG with a median sum of double bonds for the 3 acyl moieties of 7 and 2, respectively (non-parametric Wilcoxon test’s *p*-value = 1.062 × 10^−13^).
(8) Cholesteryl esters (CE): decreased concentration of 7 CE molecules with only one having a saturated acyl moiety and the remaining 6 having acyl moieties with at least 2 double bonds, of which one is an acyl moiety of 20 carbons in length with 4 double bonds (i.e., FA 20:4) and another 22 carbons in length with 6 double bonds (i.e., FA 22:6) (median double bond in acyl moieties of decreased CE of 3) and increased levels of 5 CE species, two of which had a saturated acyl chain, while another two had only one double bond and the other had two double bonds (median double bond in acyl moieties in increased CE of 1). The difference between the median double bonds in acyl moieties of decreased versus increased CE was also statistically significant (non-parametric Wilcoxon test’s *p*-value = 0.03985).
(9) Decreased levels of dehydroepiandrosterone sulfate (DHEAS) and the bile acid glycolithocholate (GLCA).
(10) Ceramide and hexosylceramides: 7 ceramides and 17 hexosylceramides, of which 10 monohexosylceramides, 6 dihexosylceramides, and 1 trihexosylceramide were found to be all relatively diminished in the stroke group.
(11) Sphingomyelins (SM): diminished concentration of 6 sphingomyelin species and 4 hydroxysphingomyelin molecules.
(12) Lysophosphatidylcholines (LPC): reduced levels of 7 LPCs and increased levels of the less abundant LPC 28:1.
(13) Phosphatidylcholines (PC): diminished concentration of 20 PC species with a median number for the sum of the double bonds of the two acyl moieties of 4 and increased concentration of 20 PC species with a median number for the sum of the double bonds of the two acyl moieties of 2 (non-parametric Wilcoxon test’s *p*-value = 0.01945).

**Figure 4:**
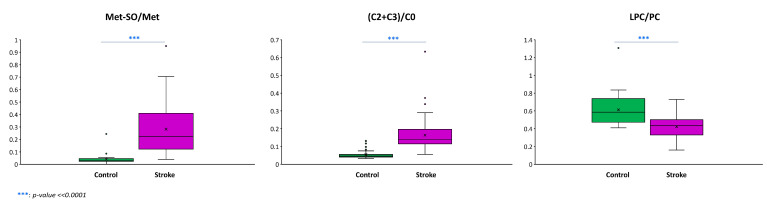
Box plots for the ratios methionine sulfoxide over methionine (Met-SO/Met), acetyl plus propionyl carnitines over free carnitine ((C2 + C3)/C0), and between the sum of lysophosphatidylcholine and the sum of phosphatidylcholine species (LPC/PC). The Met-SO/Met ratio measures oxidative stress and was significantly higher in the plasma of stroke patients; fatty acid beta-oxidation as measured by (C2 + C3)/C0 ratio was higher in stroke patients; the opposite was observed for plasmatic phospholipase A2 activity with a decreased LPC/PC in the stroke group.

Three ratios: (1) methionine sulfoxide over methionine (Met-SO/Met); (2) the sum of the two most abundant short-chain acylcarnitines acetyl and propionyl carnitines over free carnitine ((C2 + C3)/C0); and (3) the sum of all lysophosphatidylcholine over the sum of all phosphatidylcholines (Lyso-PC/PC), differed significantly between control and stroke patients (Figure 4).

The Met-SO/Met ratio is known to positively correlate with oxidative stress. The median in the stroke and control groups was 0.22 and 0.03, respectively (*p*-value << 0.0001).

The (C2 + C3)/C0 ratio is known to measure overall β-oxidation activity. The median was significantly increased in the stroke group compared with the control group (0.05 vs. 0.14, *p*-value << 0.0001).

Lyso-PC/PC is an indicator of phospholipase A2 (PLA2) activity. The activity of this enzyme was significantly decreased in the stroke group compared to the activity in the control group (0.43 vs. 0.59, *p*-value << 0.0001).

## 4. Discussion

Our plasma metabolome analysis performed on 79 West African patients who had recently suffered an ischemic stroke failed to evidence a metabolomic profile specific to cryptogenic stroke compared with strokes with a well-identified etiology. This means that cryptogenic strokes are not pathophysiologically different from other causes of ischemic strokes, but simply reflect a lack of sensitivity of clinical and medical imaging to identify the primary cause of ischemia.

However, grouping all patients of determined and undetermined groups together and comparing them to healthy controls revealed a highly discriminant metabolomic signature involving more than half (234/456, 51%) of the accurately measured metabolites. Of these 234 discriminant metabolites, 100 had increased blood concentrations and 134 had decreased blood concentrations in stroke. Several of these discriminating metabolites have already been sparsely described in various previous studies, which support the reliability of our results, while several others were identified in our study for the first time in stroke.

The increase in the concentration of five **acylcarnitines** in the blood of patients is one of the hallmarks of our stroke signature. In a metabolomic study undertaken in China in patients who had suffered an acute ischemic stroke, it was shown that increased acylcarnitines were a major discriminator in patient blood compared with controls [13]. All acylcarnitines that increased in our study (C2, C3, C4, C18:1, and C18:2) were also reported as increased in this article. A review article also listed several studies showing this increase in acylcarnitines in the blood of ischemic stroke patients [8]. Acylcarnitines are the mode of transport of fatty acids for their oxidation through mitochondrial fatty acid oxidation, and their accumulation in the blood indicates that acute ischemic stroke is accompanied by energetic stress. This is reinforced by the fact that the (C2 + C3)/C0 ratio, measuring overall β-oxidation activity, was significantly increased in the stroke group compared with the control group (*p*-value < 0.0001).

Another hallmark of our signature is the increase in **methionine sulfoxide** and **Met-SO/Met ratio,** which are both known to correlate with oxidative stress. Methionine sulfoxide is an oxidized form of methionine that is also involved in one-carbon metabolism. This increase in methionine sulfoxide following a stroke has not yet been reported. One study conducted on two large cohorts of women showed that blood methionine sulfoxide was one of the four metabolites most significantly correlated with an increased risk of ischemic stroke [14]. It could therefore already exist at increased levels in these at-risk patients, but the acute ischemic stroke itself could cause this increase. Methionine oxidation in proteins has also been shown to be a major determinant of post-ischemic injury mediated by NF-kappaB activation, leading to neutrophil recruitment and neurovascular inflammation in acute ischemic stroke in a murine model [15]. Thus, this dramatic increase in methionine sulfoxide in stroke patients could be a biomarker of at-risk patients but also a poor prognostic factor for post-stroke recovery.

**Homocysteine** is a sulfur amino acid that is also derived from methionine metabolism and involved in one-carbon metabolism. Homocysteine is a well-known pejorative factor, with pro-oxidant properties, in cardiovascular diseases, which has frequently been linked to ischemic stroke. A recent meta-analysis and systematic review confirms that ischemic stroke patients have significantly higher homocysteine levels than controls [16]. The increase in this biomarker therefore constitutes a good internal quality control for our study.

**Trigonelline** is an alkaloid derived both from endogenous nicotinic acid metabolism (involved in NAD metabolism and thus in the energetic metabolism) and from food (supplied by plants), which has never been shown to be modified in stroke. However, its antioxidant neuroprotective action has been demonstrated in an ischemic stroke model in rats [17]. Its decrease in patients who had suffered a stroke could therefore be linked to oxidative stress.

**Polyamines** (putrescine, spermine, and spermidine), which are involved in various cell signaling pathways, were all found to be increased in the blood of patients who had suffered an acute ischemic stroke. The increase in polyamines in neuronal injury following cerebral ischemia has already been reported [18]. Polyamine biosynthesis is increased by cerebral ischemia through the induction of ornithine decarboxylase, a key enzyme in the polyamine biosynthetic pathway [19]. Polyamines are also linked to glutamate-mediated neurotoxicity because they can bind to the N-methyl-D-aspartate (NMDA)-sensitive subtype of glutamate receptors to potentiate cellular responses to glutamate [19].

**1-Methylhistidine** is an amino acid derivative, which unlike other amino acids was found to have an increased blood concentration in stroke. It has never been associated with stroke before, except for its ability to differentiate between depressed and non-depressed post-stroke patients [20] and to differentiate between strokes due to an occlusion of large vessels and those due to an occlusion of small vessels [21].

There is an abundance of literature concerning the disturbance and the role of neurotransmitters in the stroke, recovery from the stroke, its sequelae, and the mood disorders that can follow. The increase in **dopamine**, **serotonin**, and **GABA** (γ-aminobutyric acid) in stroke patients in our study testifies to this acute disturbance that no metabolomic study has reported before to our knowledge. Interestingly, **α-aminobutyric acid**, which is an isomer of GABA, was also increased in our stroke cohort.

**Dehydroepiandrosterone sulfate (DHEAS)** is an intermediate in the metabolism of dehydroepiandrosterone (DHEA). It is an endogenous androgen steroid that is produced by the adrenal cortex. DHEAS is itself a neuroprotective neuro-hormone that modulates the action of glycine, NMDA, and some GABA receptors. Interestingly, low levels of this compound have been shown to be associated with an increased risk of ischemic stroke in women [22], but to our knowledge, its lowering has never been reported in stroke patients.

**α-aminoadipate** is an intermediate of the lysine synthesis pathway and is also a biomarker of insulin resistance [23]. No study on strokes has previously shown it to increase, which could be attributed to pre-existing insulin resistance in the group of patients who had suffered a stroke of determined origin (see Table 1).

Our stroke signature also shows a decrease in the concentration of the related metabolites **choline**, **betaine**, **Pro-betaine**, and **Trp-betaine**. Choline is a quaternary ammonium hydroxide derivative. It is one of the main sources of methyl groups in the body. It is an essential nutrient that plays roles in cell membrane structure as a precursor of phosphatidylcholine, which is a major component of cell membranes. The decreased concentration of choline was already found in patients with carotid stenosis, indicating that this reduced concentration could at least in part already exist prior to stroke as a pejorative vascular factor [24]. Choline is oxidized to betaine and is a substrate in the betaine–homocysteine methyltransferase reaction, thus linking choline and betaine to the alteration of one-carbon metabolism, which is known to be altered in cases of stroke [8]. Since choline and betaine have been associated with a reduced risk of cardiovascular events and recurrent stroke [25], their decrease in our study is a pejorative factor that could either pre-exist or be provoked by stroke.

To our knowledge, no study has previously reported a decrease in **sarcosine** concentration in cases of stroke. Sarcosine is formed from choline consumption and methionine metabolism and is rapidly degraded to glycine, all of which were decreased in our signature. This decrease in sarcosine is therefore probably also related to the alteration of folate-dependent one-carbon metabolism.

**Beta-alanine** is a neurotransmitter capable of acting on certain GABA receptors and the NMDA receptor [26]. It has been shown to have a neuroprotective role during cerebral ischemia [27]. Its decrease in our signature is therefore a pejorative factor.

**Hippuric acid,** which was found to be diminished in stroke, is an acyl-glycine produced by the conjugation of benzoic acid and glycine, which is an aromatic compound from diet found in urine. We did not find any articles reporting the modification of its blood concentration in ischemic stroke.

To our knowledge, the decrease in **xanthine** has never been reported in previous metabolomic studies of stroke. But xanthine oxidase, which converts hypoxanthine to xanthine and then xanthine to uric acid, has been linked to stroke. Increased activity of this enzyme in the blood is a risk factor for stroke onset and a prognostic factor for stroke [28], and its enzymatic activity in saliva has also been shown to be a potential biomarker of stroke [29]. This enzyme is a superoxide-generating enzyme and therefore generates oxidative stress.

**3-Indolepropionic** is produced from tryptophan by the intestinal microbiota. Its neuroprotective action through its antioxidant effect has previously been demonstrated [30], as well as its ability to reduce the effects of ischemic stroke in mice [31]. The decrease in the concentration of this compound found in our signature establishes a potential link with the microbiota and the decrease in antioxidant protection in stroke. Its beneficial effect on cerebral injury, mediated by the gut microbiota, has been shown in a mouse model of cerebral artery occlusion [31].

**Glycolithocholic acid sulfate (GLCA)** is a glycine-conjugated bile acid formed by hepatocytes and intestinal microbiota. To our knowledge, an alteration of its concentration has never been shown in stroke, but tauroursodeoxycholic acid (TUDCA), a related metabolite, has been shown to have a neuroprotective effect in acute stroke in rats [32].

**Docosahexaenoic acid** (DHA, 22:6 ω3) is an omega-3 polyunsaturated fatty acid. It is provided through one’s diet or by endogenous synthesis from eicosapentaenoic acid, which is itself synthesized from α-linolenic acid. DHA is the most abundant omega-3 fatty acid in the brain and has neuroprotective and anti-inflammatory properties. DHA prevents ischemic-stroke-induced neurological damage [33]. This component is one of the most discriminating of our signature, and its decrease is therefore a pejorative factor in stroke.

**Arachidonic acid** (20:4(ω-6)) is an omega-6 polyunsaturated fatty acid. It is present in phospholipids, notably phosphatidylcholines, which make up the body’s cell membranes. It is involved in cell signaling as a secondary messenger, regulating key enzymes, such as phospholipases. It is also a key intermediary in inflammation and can act as a vasodilator. Arachidonic acid can be released from a phospholipid molecule by phospholipase A2 (PLA2) and from a diglyceride molecule by diacylglycerol lipase. Its involvement in ischemic stroke is well known [34].

Our stroke signature also shows extensive lipidomic remodeling, involving 40 **phosphatidylcholines** (20 increased and 20 decreased), 8 **lysophosphatidylcholines** (7 decreased and 1 increased), 6 **sphingomyelins** (all decreased) and 4 **hydroxysphingomyelins** (decreased), 7 **ceramides** and **hexosylceramides** (all decreased), 12 **cholesterol esters** (7 decreased and 5 increased), and 88 **diacylglycerols** (28 decreased and 57 increased), as well as 4 **diacylglycerols** (3 decreased and 1 increased). This lipid remodeling is probably due to cell destruction, as most of these lipids are components of cell membranes. Previous metabolomic studies have already reported different variations in the concentration of these lipid species [10].

Another feature of our signature is the change in the **degree of saturation of the fatty acids** present in these complex lipids. We noted a significant overall increase in the saturation of fatty acids present in complex lipids (cholesterol esters, triacylglycerols, and phosphatidylcholines) during strokes, which means that there was a general loss of double bonds carried by fatty acids during strokes. In other words, there is a general shift from polyunsaturated to monounsaturated fatty acids, which are less oxidizable [35]. As these double bonds are sensitive to oxidative stress, the increased saturation of these fatty acids could be a response to the oxidative stress induced by strokes.

There are several limitations to our study. The sample size and heterogeneity of the patient population studied may not have been sufficient to detect a distinct metabolic profile for cryptogenic strokes. The patients who had recently suffered a cryptogenic stroke may have had a wide range of underlying causes, making it difficult to identify a unique metabolic signature. The lack of a distinct metabolic profile for cryptogenic strokes may also be due to technical limitations in the metabolomic analysis. The sensitivity and biochemical coverage of the analytical approach may not have been high enough to detect subtle differences in metabolite profiles between the different stroke subtypes. Further studies with larger sample sizes and greater metabolite coverage may be helpful in elucidating potential specific metabolic pathways involved in cryptogenic strokes.

## 5. Conclusions

The metabolomic exploration of ischemic strokes reported here failed to identify any specific signature for cryptogenic strokes, suggesting an absence of specific pathophysiological mechanisms. However, when compared to controls, all strokes revealed a highly discriminant metabolic signature involving more than half of the metabolites explored. The comparison of this signature with data from the literature enabled the identification of eight main metabolic alterations and a dozen metabolites, which had never been associated with strokes before (synthetic summary in Table 2). Overall, this signature highlights oxidative and energetic stress, alteration of monocarbon metabolism, neurotransmission, and intestinal microbiota, as well as reduced neuroprotection and inflammation. Interestingly, some of these metabolic alterations could constitute potential therapeutic targets aimed at mitigating the consequences of a stroke, for example, by combating oxidative stress, energetic deficiency, and inflammation. Several features identified here are also candidate biomarkers that are likely to facilitate more personalized patient care, such as the three ratios that we have highlighted in this article, making it possible to assess oxidative stress, energy deficit, and phospholipase A2 activity. This search for biomarkers and personalized therapies would therefore greatly benefit from the future development of metabolomics, which will have to be conducted on larger cohorts including the different stroke subtypes.

## Figures and Tables

**Table 1:** General features of the groups studied.

	Cryptogenic Stroke (*n* = 40)	Determined Stroke (*n* = 39)	Controls (*n* = 40)	*p*-Value
Epidemiological parameters				
Mean age (years)	47 ± 11	57.02 ± 14.91	47 ± 12	<0.001
Sex ratio	1.35	1.35	1.35	-
Biological parameters				
Total cholesterol (mmol/L)	5.08 ± 1.78	4.98 ± 1.78	5.06 ± 0.80	0.904
HDL-c (mmol/L)	1.19 ± 0.33	1.14 ± 0.33	1.52 ± 0.25	0.353
LDL-c (mmol/L)	2.71 ± 1.26	2.68 ± 1.21	3.33 ± 0.62	0.762
Triglycerides (mmol/L)	0.98 ± 0.44	1.04 ± 0.54	0.89 ± 0.41	0.725
Blood glucose (mmol/L)	5.3 ± 0.99	6.6 ± 2.03	-	<0.001
Urea (mmol/L)	3.7 ± 2.66	4.80 ± 2.81	2.7 ± 0.63	0.007
Creatinine (µmol/L)	65.68 ± 13.61	66.12 ± 25.72	70.54 ± 10.96	0.254
Uric acid (µmol/L)	332.9 ± 73.75	337.6 ± 128.47	286 ± 53.53	0.912
CRP (nmol/L)	32.47 ± 24.76	409.52 ± 620.96	18.38 ± 18.09	<0.001
AST (µkat/L)	0.57 ± 0.26	0.97 ± 0.99	0.39 ± 0.09	0.012
ALT (µkat/L)	0.42 ± 0.39	0.50 ± 0.65	0.24 ± 0.15	0.123
GGT (µkat/L)	1.01 ± 0.97	1.70 ± 3.13	0.54 ± 0.24	0.080
ALP (µkat/L)	1 ± 0.35	1.50 ± 1.50	-	0.026
Clinical and lifestyle parameters				
HTA (%)	45	84.61	-	0.002
Diabetes (%)	15	61.53	-	0.040
Dyslipidemia (%)	22.50	64.10	-	0.012
Sedentary lifestyle (%)	25	51.28	-	<0.001
Smoking (%)	25	17.94	-	<0.001
Alcoholism (%)	15	2.56	-	<0.001
Drugs (%)	5	-	-	-

**Table 2:** Main altered metabolic pathways related to the discriminating metabolites of the blood ischemic stroke signature, and new stroke-related metabolites.

Related to oxidative stress	Methionine sulfoxide, Met-SO/Met ratio, homocysteine, trigonelline, spermine, spermidine, putrescine, amino acids, α-aminoadipate, xanthine, 3-Indolepropionic, overall increase in the saturation of fatty acids present in complex lipids
Related to energetic crisis	Acylcarnitines C2, C3, C4, C18:1, and C18:2 (C2 + C3)/C0 ratio, trigonelline, amino acids
Related to cell membrane damage	Choline, betaine, Pro-betaine, and Trp-betaine, docosahexaenoic acid, phosphatidylcholines, lysophosphatidylcholines, sphingomyelins, hydroxysphingomyelins, ceramides, hexosylceramides, cholesterol esters, diacylglycerols, diacylglycerols
Related to one-carbon metabolism	Methionine, methionine sulfoxide, homocysteine, choline, betaine, Pro-betaine, and Trp-betaine, sarcosine
Related to neurotransmission and cell signaling	Spermine, spermidine, putrescine, amino acids, dopamine, serotonin, GABA, α-aminobutyric acid, choline, beta-alanine, dehydroepiandrosterone sulfate, α-aminobutyric acid, arachidonic acid
Gut microbiota and or diet-related metabolites	Trigonelline, hippuric acid, 3-indolepropionic, glycolithocholic acid sulfate (GLCA), docosahexaenoic acid (DHA)
Related to inflammation	Methionine sulfoxide, homocysteine, docosahexaenoic acid (DHA), arachidonic acid
Decreased concentration of neuroprotective metabolites	Trigonelline, beta-alanine, 3-indolepropionic, glycolithocholic acid sulfate (GLCA), dehydroepiandrosterone sulfate (DHEAS), docosahexaenoic acid (DHA)
New metabolites related to acute ischemic stroke	Methionine sulfoxide, Met-SO/Met ratio, trigonelline, 1-methyl-histidine, dopamine, serotonin, GABA, α-aminobutyric acid, α-aminoadipate, sarcosine, hippuric acid, xanthine, glycolithocholic acid sulfate (GLCA), dehydroepiandrosterone sulfate (DHEAS)

## Data Availability

All experimental datasets used throughout the present study are available on reasonable request to the corresponding author. All raw metabolomics data are given in Supplementary Table S1.

## Supplementary Materials

The following supporting information can be downloaded at https://www.mdpi.com/article/10.3390/antiox13010060/s1, Figure S1: Batch effect normalization; Table S1: Raw data: 56,986 plasma concentrations in μM of 456 metabolites from 119 participants and pools; Table S2: *p*-values, loadings, and fold changes of the 234 metabolites with significant modified concentrations in stroke vs. controls.
