# Supplementary material for: A Metabolomic Signature of Ischemic Stroke Showing Acute Oxidative and Energetic Stress
Source: Antioxidants (Basel). 2023 Dec 29;13(1):60. doi: 10.3390/antiox13010060 (PMC10812657; doi:10.3390/antiox13010060)
Supplement: Supplementary file 1 [file antioxidants-13-00060-s001.zip › Supplementary Figure S1.pdf]

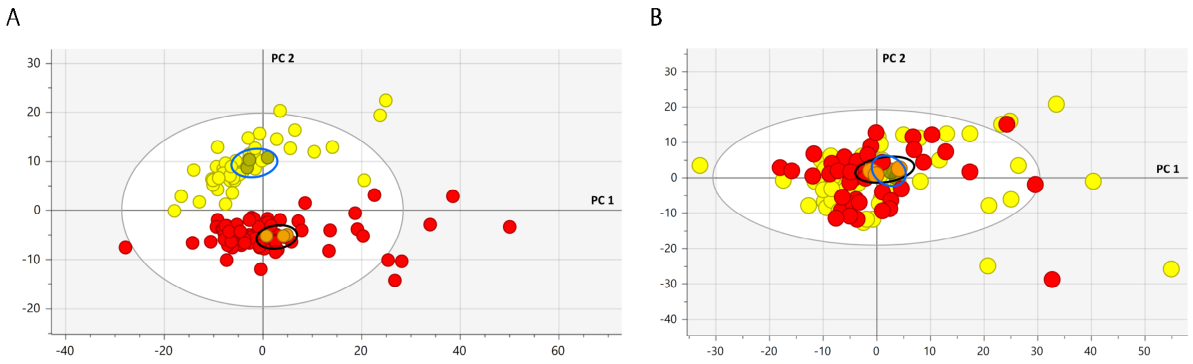

Supplementary Figure S1: Batch effect normalization. **A**-Without batch correction, a marked batch effect appeared in principal component analysis (PCA) with a neat discrimination between samples analyzed in the first batch/kit (yellow circles) and those analyzed in the second batch/kit (red circles). Pool samples (green and orange circles delimited by blue and black ellipses, respectively) seem also seem to represent samples on each batch very well. **B**- After batch correction, patient and pool samples no longer segregate according to the batch factor. No predictive supervised (OPLS-DA) model separating samples according to batch effect was found after normalization.
